# Swertiamarin: An Active Lead from *Enicostemma littorale* Regulates Hepatic and Adipose Tissue Gene Expression by Targeting PPAR-**γ** and Improves Insulin Sensitivity in Experimental NIDDM Rat Model

**DOI:** 10.1155/2013/358673

**Published:** 2013-06-09

**Authors:** Tushar P. Patel, Sanket Soni, Pankti Parikh, Jeetendra Gosai, Ragitha Chruvattil, Sarita Gupta

**Affiliations:** Department of Biochemistry, Faculty of Science, The Maharaja Sayajirao University of Baroda, Vadodara, Gujarat-390 005, India

## Abstract

*Enicostemma littorale* (EL) Blume is one of the herbs widely used for treating and alleviating the effects of both type I and type II diabetes. However, lack of understanding of mechanism precludes the use of the herb and its molecules. In this study, we attempt to unravel the molecular mechanism of action of swertiamarin, a compound isolated form EL, by comparing its molecular effects with those of aqueous EL extract in alleviating the insulin resistance in type II diabetes. We further investigated hypolipidemic and insulin sensitizing effect of swertiamarin in experimentally induced noninsulin dependent diabetes mellitus (NIDDM) in rats. Swertiamarin (50 mg/kg) and aqueous extract (15 grams dried plant equivalent extract/kg) were administered to rats orally for 40 days and tight regulation of serum glucose, insulin, and lipid profile was found in both groups. Their mode of action was by restoring G6Pase and HMG-CoA reductase activities to normal levels and restoring normal transcriptional levels of PEPCK, GK, Glut 2, PPAR-**γ**, leptin, adiponectin, LPL, SREBP-1c, and Glut 4 genes. This suggests that both treatments increased insulin sensitivity and regulated carbohydrate and fat metabolism. This is the first report on the role of SM in regulating the PPAR**γ**-mediated regulation of candidate genes involved in metabolism in peripheral tissues *in vivo*.

## 1. Introduction 

Diabetes mellitus is the third most prevalent fatal disease in the world. Epidemiology shows that it is one of the major global health problems in the current scenario, targeting 6.4% of total world population. Around fifty million people are diabetic in Indian subcontinent; hence, India leads globally in this disease [1]. Indian population is more prone to diabetes than western population as their metabolism is quite different with lots of epigenetic modifications. Constant migration of people from rural to urban areas has contributed significantly in availability of food, calorie intake, and physical activities, which has major impact on the metabolic programming of an individual [2]. To understand the physiological and metabolic alterations of this disorder, many animal models are used. NA-STZ rat is a nonobese type 2 diabetes model that reflects the majority of diabetic patients among Asian races [3]. 

Type II diabetes mellitus is a heterogeneous metabolic disorder. Liver, skeletal muscles, and adipose tissues being insulin sensitive tissues show significant metabolic changes [4]. Insulin, apart from governing glucose uptake and metabolism, also influences the expression level of many genes related to energy metabolism [5].

Glut 2 plays a major role in glucose uptake and its metabolism in hepatic tissue, whose gene expression is influenced by glucose and insulin concentrations; hence, remarkable alterations in glucose would lead to significant change in the expression profile of this transporter. In diabetic condition, the expression of glucokinase (GK) enzyme is downregulated, which eventually leads to insulin resistance and hyperglycemia [6, 7]. PEPCK is a rate-limiting enzyme in gluconeogenesis; hence, its elevated expression leads to increased hepatic glucose production (HGP). The processes of glycolysis, gluconeogenesis, glycogenesis, and glycogenolysis are governed by insulin action, which are hampered due to altered action of insulin in TIIDM [8]. 

Obesity and dyslipidemia lead to the development of type II diabetes. Secretion of adipokines play a key role in controlling glucose and fat homeostasis of the entire body. PPAR-**γ**, playing a major role in regulation of transcription, is responsible for adipogenesis, mature adipocyte function, insulin signaling, carbohydrate metabolism, fat metabolism, and secretion of various adipokines like adiponectin, leptin, and so forth. Adiponectin positively and leptin negatively regulate insulin signaling in liver and muscle tissues [9]. Looking at the importance of this regulator, most studies have been directed toward developing synthetic PPARs ligands for insulin resistance and dyslipidemia in amelioration of TIIDM complications [10].

There are many treatments available ranging from synthetic drugs, like metformin, thiazolidinediones, GLP-1, DPP-4 inhibitor, and so forth, to herbal formulations like *Momordica charantia*, *Artemisia dracunculus*, *Gymnema sylvestre*, and so forth in amelioration of obesity and TIIDM [11]. In recent years, there has been renewed interest in the treatment of diabetes using herbal drugs, as World Health Organization (WHO) has recommended evaluation of the effectiveness of plants due to side effects of modern drugs [12]. Our lab has well documented *Enicostemma littorale* Blume owing antioxidant, hypolipidemic, and antidiabetic activities both in animals and human diabetic patients [13, 14]. Apart from antidiabetic activity, islet neogenic property of swertisin and normoglycemia in a diabetic rat are also reported [15]. Hence, it can be presumed that the EL extract potentially owes varied beneficiary activities due to the presence of many compounds within it. Swertiamarin (SM) is the major compound found in EL Blume and its pharmacokinetic study suggest that it is rapidly distributed in most of the tissues. Among all the tissues, the highest concentrations were found to be absorbed in the liver and its elimination was through kidney [16]. 

Many studies have been done to explore the mechanistic action of SM on TIIDM but are restricted to in vitro only. Also, other groups have focused on physiological and biochemical studies in neonatal-STZ models to understand role of SM for the treatment of obesity and dyslipidemia. However, the mechanism of action of SM has not been explored at a systemic level [17, 18].

Therefore, for the first time in this study we aim to assess the antidiabetic efficiency of SM in treating nicotinamide-streptozotocin (NA-STZ) diabetic rats and to elucidate its probable mechanisms of action. The current study was designed to answer the key question, “what is the mechanism of SM in regulating the expression levels of the candidate genes involved in carbohydrate, fat metabolism and insulin signaling in the liver and adipose tissue in TIIDM?”

## 2. Materials and Methods

### 2.1. Plant Material, Preparation of Aqueous EL Extract, and Isolation of Swertiamarin

**Figure 1:**
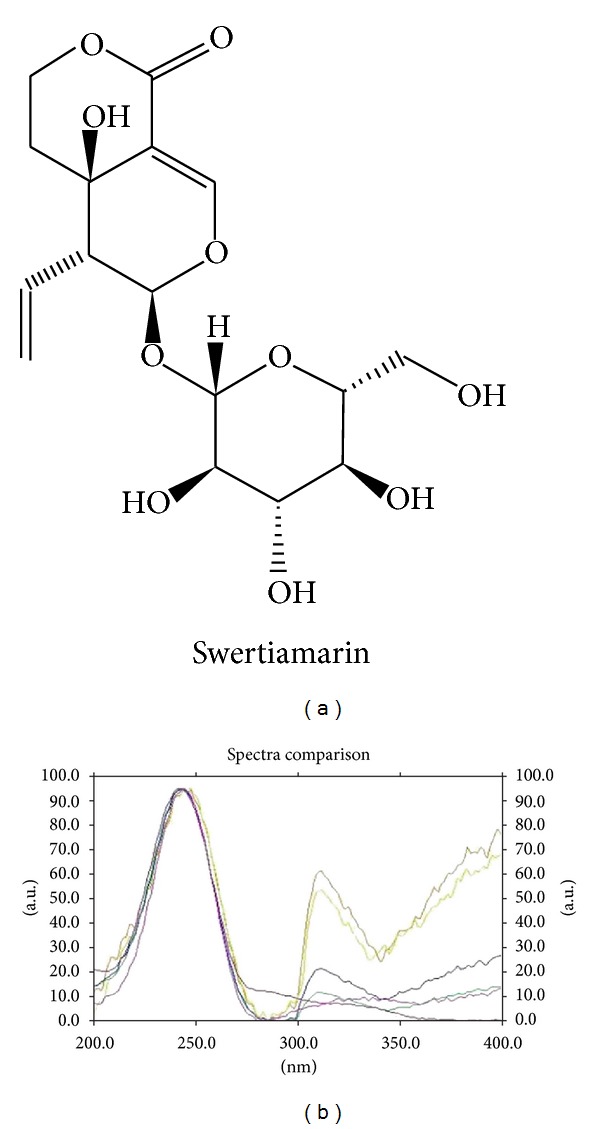
(a) Chemical structure of swertiamarin. (b) Overlay of ultraviolet absorption spectrum of swertiamarin isolated in lab and reference standard (*λ*max: 240–245 nm).

The plant material of dry *E. littorale* was procured from Saurashtra region, Gujarat, India, during the month of August. Specimen was authenticated at Botany Department, M. S. University, Baroda with Voucher Specimen number [Oza 51,51(a)] deposited at the Herbarium of Botany Department, M. S. University, Baroda. Whole plant material was cleaned and dried. The fine powder of 40–60 mesh particle size was prepared in an electric grinder. The powder was soaked in thrice the amount of water for 2 hours and then boiled for 30 minutes. Three such extractions were done from each batch. Residue was removed by filtration, and water-soluble filtrate was pooled and evaporated to obtain extract concentration of 1 g dry plant weight equivalent per mL as per the method described above [19]. The yield of dry EL extract was found to be 28% (w/w). Isolation and characterization of swertiamarin from *E. littorale* were carried out by recording melting point and UV spectrometry with the standard sample of swertiamarin (Figure 1(a)). Purity of the sample was checked by HPTLC on ethyl acetate: methanol : water (0.7 : 0.2 : 0.1) as a solvent system [20].

### 2.2. Animals and Housing

Male *Charles Foster* rats housed at animal house facility of Department of Biochemistry were used for the study with *ad libitum* access to water and commercial chow (Pranav Agro Industries Ltd, Pune, India) in a well-ventilated animal unit (26–28°C, humidity 60%, 12 h light—12 h dark cycle). Care and procedures adopted for the present investigation were in accordance with the approval of the Institutional Animal Ethics Committee (938/a/06/CPCSEA, BC/14/2009-10). NIDDM rat model was developed by intraperitoneal injection of nicotinamide dissolved in normal saline at a concentration of 230 mg/kg body weight 15 minutes before giving an intraperitoneal injection of streptozotocin (Sigma, Aldrich) which was dissolved in 0.1 M citrate buffer (pH 4.5) at a concentration of 65 mg/kg body weight [21]. Hyperglycemia was confirmed by the elevated glucose level in fasting and postprandial blood sugar (PP_2_BS) at 15–20 days of streptozotocin-nicotinamide injection.

### 2.3. Dosing of Swertiamarin and Aqueous Extract in NIDDM Rats

Swertiamarin and aqueous extract were orally administered for 40 days, and fasting serum glucose levels, OGTT profiles, and serum triglyceride levels were monitored. Rats were divided into five groups having six rats in each group; group I: normal control (NC), group II: DM, group III: DM + Aqueous extract (15 grams dried plant equivalent extract/kg b.w/day, p.o.), group IV: DM + swertiamarin (50 mg/kg/day, p.o.), and group V: DM + metformin (500 mg/kg b.w/day, p.o.). 

### 2.4. Biochemical Parameters

#### 2.4.1. Oral Glucose Tolerance Test (OGTT), Serum Insulin, and Lipid Profile

Rats were kept for overnight (10–12 hrs) fasting, and blood was collected from retro orbital sinus for estimation of fasting blood sugar. To measure OGTT of the rats, 2 gms/kg body weight of glucose was given orally and blood was collected at regular interval of every 30 min. till 2 hours. Serum was separated and glucose level was estimated using GOD-POD method by commercially available kit (Enzopak, India). Fasting serum insulin was estimated by rat insulin ELISA kit (Mercodia, Sweden). Total cholesterol, HDL-cholesterol, and TG was estimated using commercially available kits (Enzopak, India), and then the values of LDL-cholesterol and VLDL-cholesterol were derived from Friedewald's formula.

#### 2.4.2. Determination of Liver Enzymes

Glucose-6-phosphatase was assayed according to the method of Koida and Oda, 1959, and the inorganic phosphorus (Pi) liberated was estimated by Fiske and Subbarow method, 1925. The ratio of absorbance of HMG CoA/absorbance of mevalonate was taken as an index of the activity of HMG CoA reductase activity required to convert HMG CoA to mevalonate, in the presence of NADPH [14]. 

#### 2.4.3. RNA Isolation and Semiquantitative PCR

Animals from each group were sacrificed, and tissues (liver and adipocytes) were pooled. RNA was isolated from the homogenized liver and adipose tissue using the TRIzol reagent (Sigma Aldrich) as per manufacturer's instructions. A reverse-transcription reaction was performed using 2 *μ*g RNA with MuLV reverse transcriptase in a 20 *μ*L reaction volume containing DEPC treated water (Fermentas Kit). PCR product was amplified using gene-specific primers (Table 1). *β*-Actin was used as an internal control. The PCR products were analyzed by electrophoresis on 2.0% agarose gels or 15% DNA-PAGE, the gels were photographed after staining with ethidium bromide, and intensities of the band were calculated by densitometric analysis using the Image J software. 

#### 2.4.4. Immunoprecipitation and Immunoblotting for Insulin Signaling Proteins

Tissues were collected, suspended in lysis buffer containing 1X protease inhibitor cocktail, and homogenized. After centrifugation at 16000 g for 15 min. at 4°C, the supernatant was collected. Total protein content was quantified using Bradford assay (Biorad Bradford Solution, USA). Immunoprecipitation with insulin receptor (anti-IR*β* 1 : 50) was performed using dynabeads G-protein IP kit (Invitrogen). Protein was loaded on a 10% SDS-polyacrylamide gel and then electrophoretically transferred onto a nitrocellulose membrane (GE Healthcare). The membrane was then incubated for 1 h at room temperature in blocking buffer (TBS-T containing 5% skimmed milk) and further incubated overnight with the primary antibodies for insulin receptor (1 : 1000), p-Tyr (1 : 1000), and PI(3)K (1 : 1000) at 4°C. Membrane was then washed four times with TBS-T and incubated with HRP-conjugated secondary antibodies (1 : 2500) for 1 h. Finally, membrane was developed and visualized with enhanced chemiluminescence western blotting detection system (Millipore Inc. USA).

### 2.5. Statistical Analysis

The results were analyzed using one-way analysis of variance (ANOVA) and student's *t*-test to determine the level of significance. *P* < 0.05 was considered to be significant. Results were expressed as mean ± SEM. The statistical analysis was carried out by using the Graph Pad Prism 3.0 software.

## 3. Results

### 3.1. Isolation and Confirmation of Swertiamarin from *Enicostemma Littorale* Blume

The *n*-butanol fraction yielded 7.31% w/w of swertiamarin. HPTLC densitogram confirmed identity of compound with standard swertiamarin as well as established purity of the same (Figure 1(b)). Ultraviolet absorption spectrum showed *λ*max in the range of 240–245 nm. Melting point of the compound was 190–192°C. The mass fragmentation pattern of compound represented base peak m/z of 374 representing molecular weight and m/z of 212 (M-162) a characteristic peak after removal of sugar moiety from the compound. 

### 3.2. Swertiamarin Positively Regulates Various Physical and Biochemical Parameters in TIIDM

**Figure 2:**
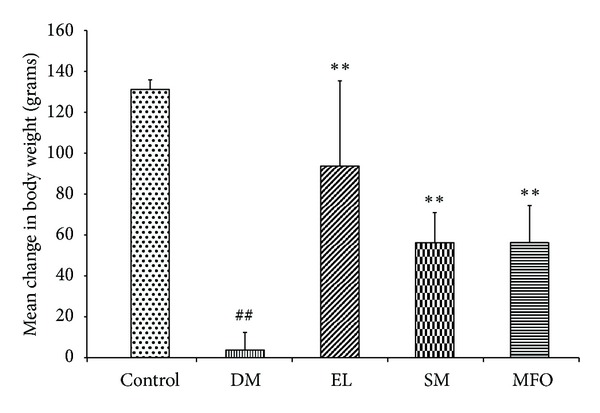
Change in body weight upon induction of diabetes and treatment of diabetic rats with EL, SM, and standard drug MFO. Data presented as mean ± SEM of 6 independent observations. ^##^ *P* < 0.05 versus control rats; **P < 0.05 versus Diabetic rats.

Decrease in body weight is a characteristic hallmark of TIIDM that happens due to the loss of the stored energy reserves. A classical way of determining the efficacy of drug treatment is the ability to restore body weight. As expected, we found a drastic decrease in the body weight in NA-STZ-induced diabetic rats as compared to the controls. The animals treated with standard drug metformin (MFO), aqueous extract, and SM showed significant increase in the body weight, which indicated reversal of DM condition (Figure 2).

**Figure 3:**
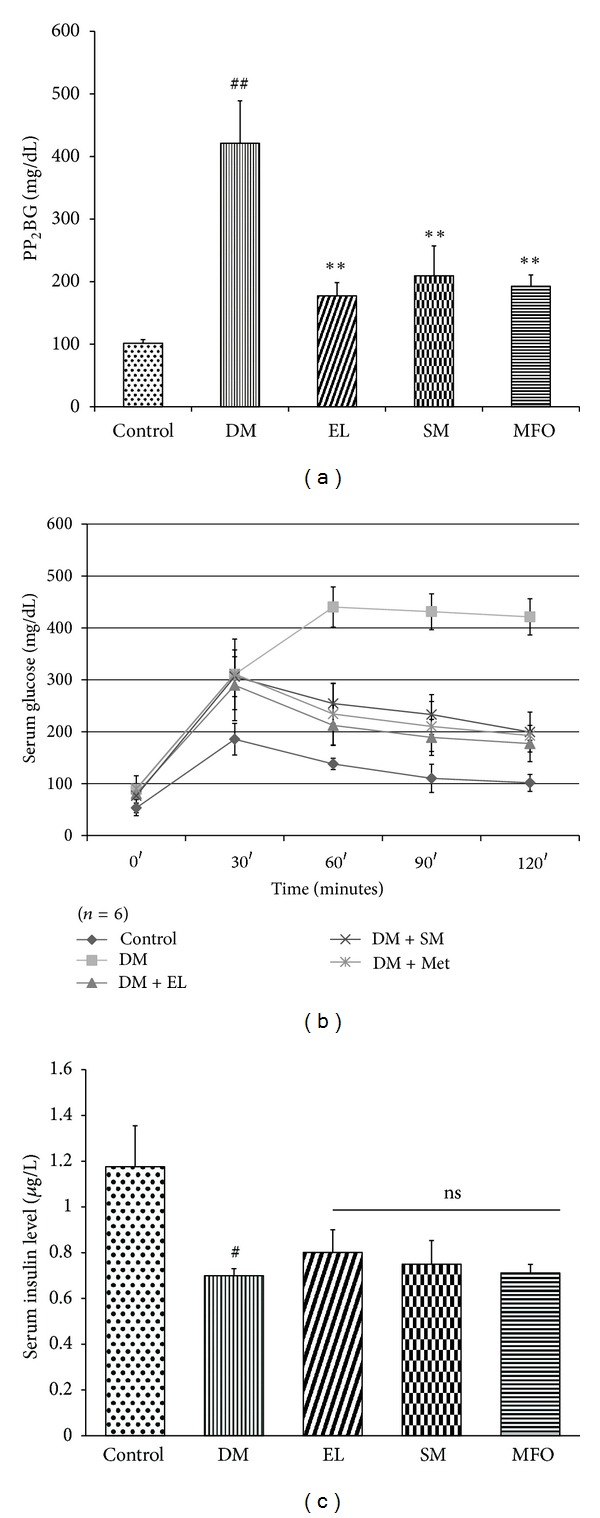
(a) Effect of EL extract, SM, and MFO treatments for 40 days on the postprandial serum glucose levels in diabetic conditions. Serum glucose levels were measured using GOD-POD. Data presented as mean ± SEM of *n* = 6 independent observations. ^##^ *P* < 0.05 versus control rats; **P < 0.05 versus diabetic rats. (b) Effect of EL extract, SM, and MFO treatments for 40 days on the OGTT profile in diabetic conditions. Serum glucose levels were measured using GOD-POD. Data presented as mean ± SEM of *n* = 6 independent observations. (c) Effect of EL extract, SM, and MFO treatments for 40 days on the serum insulin levels in diabetic conditions. Serum insulin levels were measured using ELISA kit. Data presented as mean ± SEM of 4 independent observations. ^#^ *P* < 0.05 versus control rats. *P* value ns versus diabetic rats.

Further, in an attempt to confirm the NIDDM condition in the animal model, oral glucose tolerance test was performed to ascertain severity of the diabetic condition. Our observation is in agreement with the known facts: the diabetic rats have a high PP_2_BS and showed glucose intolerance as compared to the control rats (Figures 3(a) and 3(b)). MFO effect, as expected in Type II diabetes, reduced the PP_2_BS in our rats (Figures 3(a) and 3(b)). The aqueous extract and SM-treated diabetic rats were observed to be normoglycemic.

Hyperinsulinemia is a characteristic feature of type II DM. However, in our DM group of rats, the serum insulin levels were lower than those in the control rats which matched with the reported model where insulin content was reduced up to 40% [21] (Figure 3(c)) and which mimic the later stage TIIDM. The EL extract and SM treatments were not capable of significantly ameliorating the hypoinsulinemic condition by increasing the serum insulin levels. 

### 3.3. Swertiamarin Reduces Glucose 6 Phosphatase Activity

**Figure 4:**
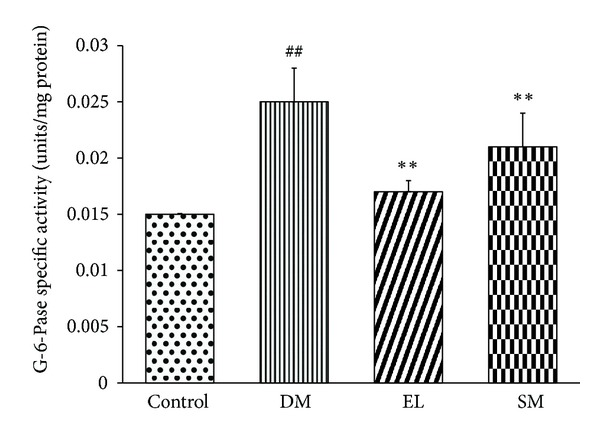
Effect of EL extract, SM, and MFO treatments for 40 days on the specific activity of G-6-Pase enzyme from hepatic tissue in diabetic conditions. It was assayed according to Koida and Oda method, and released Pi was estimated using Fiske-Subbarao method. Data presented as mean ± SEM of 5 independent observations. ^##^ *P* < 0.05 versus control rats; **P < 0.05 versus diabetic rats.

Changes due to diabetes are not only seen at the mRNA level but also at the protein levels. Many of the enzyme activities are altered in the peripheral tissues of diabetes. G-6-Pase is the key enzyme of gluconeogenesis in hepatic tissue. Its activity increases under diabetic condition due to deficiency of insulin or insulin action. The EL extract and SM restored elevated specific activity of G-6-Pase to normal levels (Figure 4).

### 3.4. Swertiamarin Regulates the Expression Levels of Candidate Genes of Carbohydrate Metabolism in TIIDM

**Figure 5:**
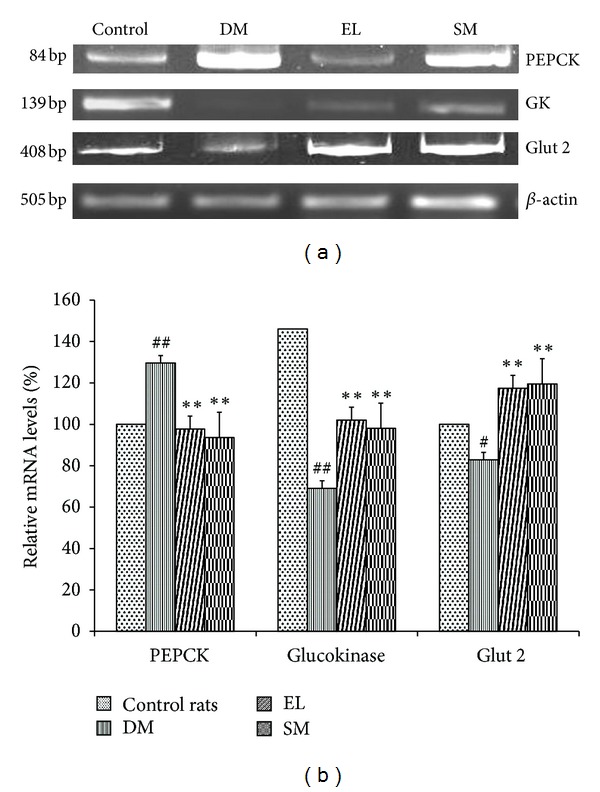
(a) Effect of EL extract and SM treatments on the mRNA expression of PEPCK, GK, Glut 2, and *β*-actin in the hepatic tissue as compared to diabetic rats (Gel image). (b) Effect of EL extract and SM treatments on the expression of PEPCK, GK, and Glut 2 in the hepatic tissue as compared to diabetic rats. The expression levels were checked using semi-quantitative RT-PCR and densitometric analysis. Data presented as mean ± SEM of 4 independent observations. ^##^ *P* < 0.05 versus control rats; **P < 0.05 versus diabetic rats.

Diabetes affects the expression of many candidate genes in the insulin dependent peripheral tissues like liver, adipose, and skeletal muscles. Glut transporters are the main glucose transporters in different peripheral tissues. Glut 2 is present in liver and is insulin independent. In diabetic rats, Glut 2 expression decreased significantly as compared to the control rats. We observed that the two treatments, EL extract and SM, rescue this decrease in expression, and the extract was more efficacious than the compound. PEPCK and glucokinase are the main enzymes of gluconeogenesis and glycolysis, respectively. They are regulated by insulin at the transcriptional level. In the diabetic condition, PEPCK has increased expression, while GK has decreased expression in liver. Treatments for diabetes should thus decrease the expression of PEPCK and increase the expression of GK. It was observed that EL extract and SM showed this (Figures 5(a) and 5(b)).

### 3.5. Swertiamarin Regulates the Altered Expression of Insulin Signaling Proteins in Liver

**Figure 6:**
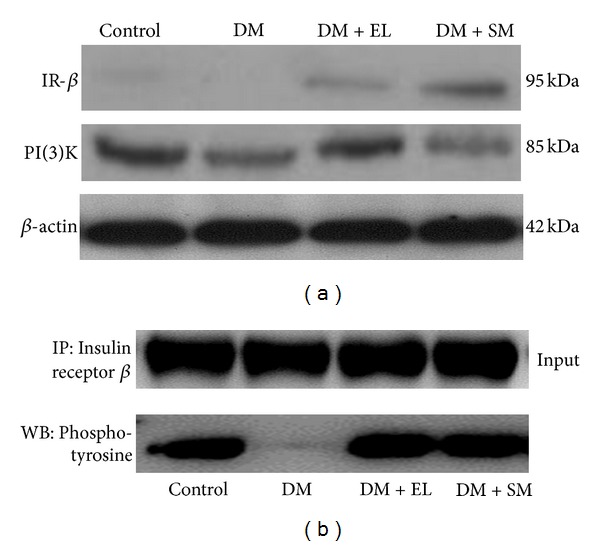
(a) Western blot study showing the effect of EL extract and SM treatments on the expression of insulin signaling proteins: IR and PI(3)K in the hepatic tissue as compared to diabetic rats. *β*-actin was taken as an internal control. (b) Immunoprecipitation study showing the effect of EL extract and SM treatments on the tyrosine phosphorylation of insulin signaling proteins: IR in the hepatic tissue as compared to diabetic rats (200 ug protein).

The liver homogenate was subjected to immunoprecipitation and immunoblotting with antiphosphotyrosine and anti-insulin receptor antibody. There was a decrease in the protein expression of insulin receptor in the diabetic group as compared to control. However, treatment with SM and EL extract restored not only the level of insulin receptor protein but also increased its phosphorylation. PI(3)K is a molecule downstream to insulin receptor, which gets recruited via IRS signaling pathway (Figures 6(a) and 6(b)).

### 3.6. Lipid Profile

We observed a significant increase in the serum triglyceride levels in the diabetic rats as compared to the control rats. This observation assertively showed that the serum triglyceride levels increase due to peripheral insulin resistance. Metformin (MFO) did not bring down the serum triglycerides levels significantly. But EL extract and its compound, SM, had a higher efficacy and reduced the serum triglyceride level near to control levels, thus making them out to be a safer alternative than the available anti-diabetic drugs. Aqueous extract and SM both were able to decrease serum cholesterol, serum LDL, and VLDL levels and increase HDL-cholesterol (Table 2).

### 3.7. Swertiamarin Regulates Activity of HMG-CoA Reductase Enzyme of Cholesterol Biosynthesis

**Figure 7:**
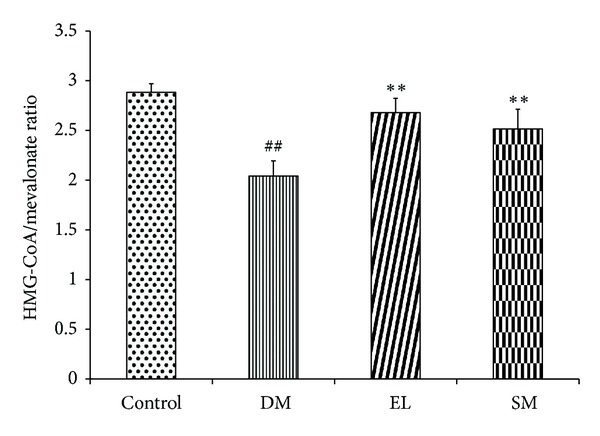
Effect of EL extract and SM treatments for 40 days on the ratio of absorbance of HMG CoA/absorbance of mevalonate was taken as an index of the of HMG CoA reductase activity from hepatic tissue in diabetic conditions. Data presented as mean ± SEM of 5 independent observations. ^##^ *P* < 0.05 versus control rats; **P < 0.05 versus diabetic rats.

HMG-CoA reductase is the major regulatory enzyme of cholesterol biosynthesis in the liver. An estimate of the enzyme activity can be used as a measure of the severity of the diabetic condition. The results of the present study showed inhibition of the HMG-CoA reductase activity in the diabetic rats treated with SM and EL extract as observed by higher HMG-Co/mevalonate (substrate/product) ratio compared to that of the diabetic control rats (Figure 7), thus supporting earlier reported hypolipidemic activity of SM.

### 3.8. Swertiamarin Regulates the Expression Levels of Various Key Enzymes of Lipid Metabolism and Glucose Transporter

**Figure 8:**
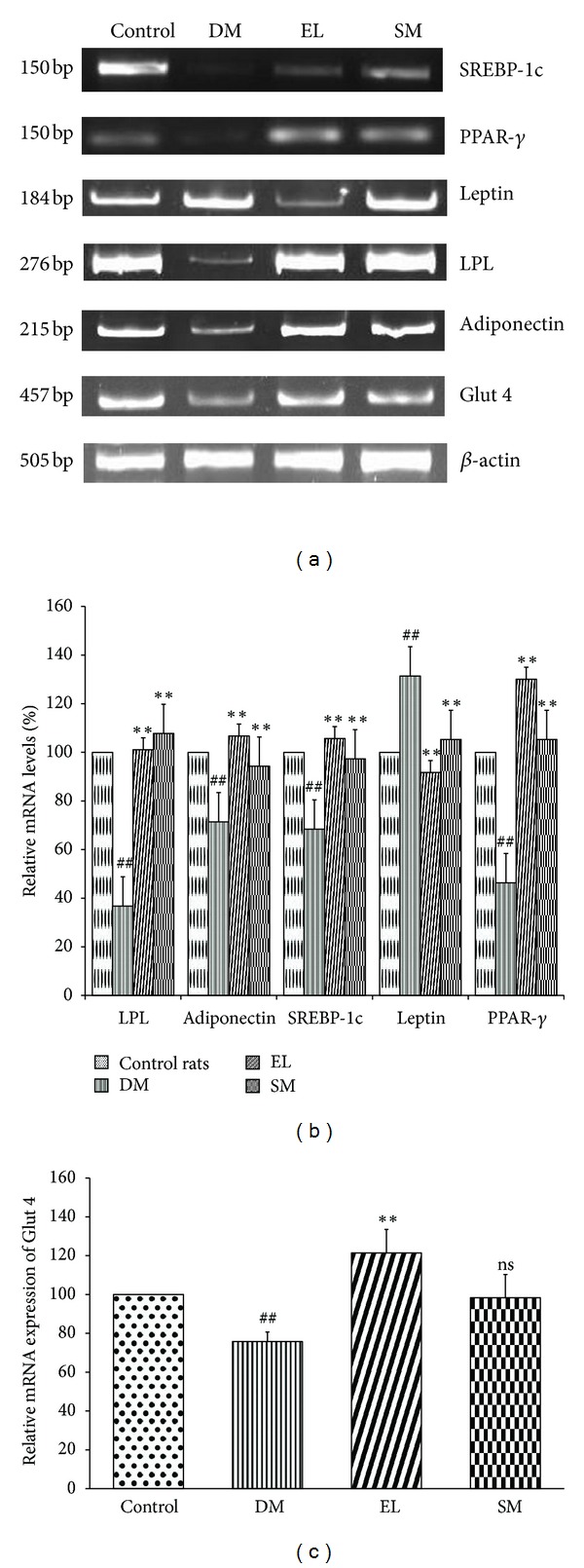
(a) Effect of EL extract and SM treatments on the mRNA expression of SREBP-1c, PPAR-*γ*, leptin, LPL, adiponectin, Glut 4, and *β*-actin in the adipose tissue as compared to diabetic rats. (Gel image). (b) Effect of EL extract and SM treatments on the expression of the major genes regulating the fat metabolism in the adipocytes as compared to diabetic rats. The expression levels were checked using semi-quantitative RT-PCR and densitometric analysis. Data presented as Mean ± SEM of 4 independent observations. ^##^ *P* < 0.05 versus control rats; **P < 0.05 versus diabetic rates. (c) Effect of EL extract and SM treatments on the expression of Glut 4 in the adipocytes as compared to diabetic rats. The expression levels were checked using semi-quantitative RT-PCR and densitometric analysis. Data presented as mean ± SEM of 4 independent observations. ^##^ *P* < 0.05 versus control rats; **P < 0.05 versus diabetic rates; *P* value ns versus diabetic rats.

Glut 4, an insulin dependent glucose transporter present in adipocytes and skeletal muscles, has decreased expression in diabetic rats due to increased insulin resistance. The key regulators of fat metabolism like adiponectin, SREBP-1c, PPAR-*γ*, and lipoprotein lipase 1 (LPL 1) are also found to be downregulated, while leptin is upregulated. The treatments with EL extract and SM helped in overcoming the insulin resistance by restoring the above gene expressions to normal levels (Figures 8(a), 8(b), and 8(c)).

### 3.9. Expression of Insulin Signaling Proteins in Adipose Tissue

**Figure 9:**
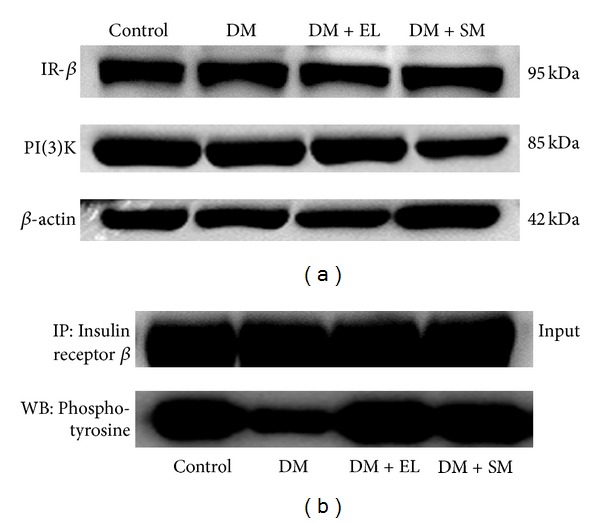
(a) Western blot study showing the effect of EL extract and SM treatments on the expression of insulin signaling proteins: IR and PI(3)K in the adipose tissue as compared to diabetic rats. *β*-actin was taken as an internal control. (b) Immunoprecipitation study showing the effect of EL extract and SM treatments on the phosphorylation of insulin signaling proteins: IR in the adipose tissue as compared to diabetic rats (100 ug protein).

The adipose tissue homogenate was subjected to immunoprecipitation and immunoblotting with antiphosphotyrosine and anti-insulin receptor antibodies. There was a decrease in the protein expression of insulin receptor and PI(3)K in the diabetic group as compared to control (Figure 9(a)). Treatment with SM and aqueous extract restored the level of insulin receptor, IR phosphorylation, and PI(3)K protein level (Figure 9(b)).

## 4. Discussion

Obesity and insulin resistance are major causes of TIIDM. Multiple problems in diabetes lead to a cascade of complications in peripheral tissues. For controlling hyperglycemia, dyslipidemia, and insulin resistance, many synthetic drugs have been used. Also, the beneficiary effects of herbal extracts and compounds have been exploited [12].

*Enicostemma litorrale *Blume belonging to Gentianaceae family has been evaluated for its hypoglycemic, antioxidant, and hypolipidemic activities [13, 14]. Further, the author's research group is continuously involved in exploring the wide spectrum hidden potentials of this plant for islet neogenesis and various diabetic complications [15, 22, 23]. Qualitative analyses of EL have demonstrated the presence of flavonoids and secoiridoid glycosides. Swertiamarin, a secoiridoid glycoside, is one of the most valuable compounds that is present in abundance and possesses various therapeutic activities: antidiabetic, antinociceptive, antilipidemic, and anti-inflammatory [17, 24]. Hence, it is interesting to unravel the mechanism of this compound's action against the development and progression of TIIDM. 

Various animal models are available for studying TIIDM. NA-STZ nonobese NIDDM rat model was selected for this study that best mimics the non-insulin dependent diabetes condition prevalent in humans [21]. Glucose intolerance, altered insulin content and skewed lipid profile of the experimental animals, resemble the hallmarks of this model that actually persist in a later stage of human TIIDM patients. Effect of SM in restoration of body weight, OGTT profile, and hypolipidemic activity on the experimental animals potentially proves the reported characteristics of this compound in the present study. Many herbal compounds are reported in regulating the expressions of the candidate genes involved in metabolic pathways and thus ameliorating TIIDM. This led to our interest in unraveling the molecular mechanism of aqueous extract and SM in restoring the altered expressions of the candidate genes involved in TIIDM.

Carbohydrate and fat metabolism regulation is governed mainly in insulin sensitive peripheral tissues like liver, muscle, and adipose tissue. Liver is the master organ in metabolism where glucokinase, PEPCK, and glycogen phosphorylase are rate limiting enzymes in glucose flux, gluconeogenesis, and glycogenolysis, respectively [8].

Decreased activity of G-6-Pase was observed in SM-treated diabetic rats, which correlate to the results reported by us previously in aqueous extract-treated diabetic rats. Reduction in the PEPCK gene expression was observed when diabetic rats were treated with SM in the current study. PEPCK expression restoration reflects increased insulin sensitivity [25–27]. Increased activity of this limiting enzyme leads to more hepatic glucose production (HGP), which worsens the diabetic condition. Glucose concentration increases the binding of SREBP-1c on promoter of Glut 2, increasing its transcription which is regulated by glucose and insulin. Expression levels of glucokinase and Glut 2 have been shown to be decreased in the hepatocytes of the diabetic rats [28]. In agreement with earlier reports, our results show that diabetic rats have decreased glucokinase and Glut 2 expressions which are reversed upon treatment with EL aqueous extract and SM. 

It is well documented that diabetic patients exhibit dyslipidemia. Our lab previously reported a decrease in serum triglycerides, cholesterol, LDL, and VLDL with increased HDL level in aqueous extract-treated cholesterol fed rats [14]. SM is beneficial in bringing the lipid profile in neonatal-STZ rats to normal. [18]. HMG-CoA reductase is a key enzyme involved in the cholesterol biosynthesis in the hepatic tissue, which increases the free fatty acid level that leads to insulin resistance. SM and aqueous extract correct lipid profile and HMG-CoA reductase activity [29]. 

Adipose tissue plays an important role in fat metabolism. In TIIDM, increased lipolysis and decreased lipogenesis occur in liver and adipose tissues. Obesity decreases expression of lipogenic genes like SREBP-1c, PPAR-*γ*, and aP2, which causes increase in hepatic lipogenesis hence leading to a fatty liver [30]. PPAR-*γ* is a key transcriptional factor regulating the expression of SREBP-1c, leptin, adiponectin, and LPL. Low adiponectin and high leptin levels can cause insulin resistance in adipocytes thus leading to diabetes [31]. In the present study, aqueous extract and SM both regulate PPAR-*γ* mRNA levels in NA-STZ-induced diabetic rat model along with induced expression of adiponectin, LPL, and SREBP-1c suggesting it as a potent modulator of diabetes-related modification in adipocytes and thus corrects overall lipid metabolism, which can correct dyslipidemia by increasing insulin sensitivity [32].

Insulin sensitivity depends on the binding of insulin to its receptor, which autophosphorylates and further leads to downstream signaling cascade. Treatment of diabetic animals with aqueous extract and SM showed increased insulin receptor protein synthesis and its autophosphorylation in liver and adipose tissues, which improves insulin sensitivity in TIIDM. Phosphorylation of PI(3)K is mainly responsible for insulin stimulated glucose uptake by Glut 4, which is responsible for peripheral glucose disposition in muscle and adipose tissue. It has been reported that cinnamon extract improves insulin action and glucose uptake by enhancing the insulin signaling pathway in skeletal muscle [33].

**Figure 10:**
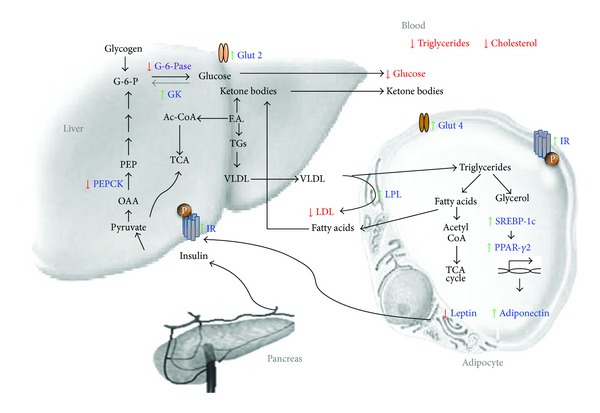
Schematic representation of swertiamarin in amelioration of insulin resistance and TIIDM. Figure shows carbohydrate and fat metabolic pathways and candidate genes which are altered during diabetes. Swertiamarin treatment modulates not only the expression of these target genes which is marked in blue but also the metabolite levels in blood marked in red.

 The results of the current study proves that SM activates PPAR-*γ* and its regulatory genes, which improves fat metabolism in adipose tissue. By controlling PPAR-*γ*, SM can maintain the status of small adipocytes that reduces expression of leptin and TNF-*α* and increases expression of adiponectin. Increased Adiponectin secretion acts in an autocrine and paracrine manner, which improves expression of insulin receptor, its autophosphorylation, and downstream insulin signaling in liver as well as in adipose tissue [34]. This is the need of the hour, a drug which is able to maintain a balance between all the players involved in the carbohydrate and fat metabolism in the peripheral tissues (Figure 10). 

## 5. Conclusion

The NA + STZ-treated rats show glucose intolerance, increased serum TG, and decreased serum insulin levels, indicating NIDDM-like condition. Treatment with aqueous EL extract and swertiamarin has been found to reduce the glycemic burden as monitored by OGTT profile. In diabetic rats, swertiamarin enhances insulin sensitivity resulting in restoration of altered gene expression of glucose metabolism in liver. In dyslipidemic condition, swertiamarin plays a crucial role in lowering surplus cholesterol by inhibiting HMG-CoA reductase activity. This is the first report *in vivo* that highlights a significant role of SM as a regulator of gene expression under the control of transcriptional factors like PPAR-*γ*, hence suggesting that SM improves insulin sensitivity and modulates carbohydrate and fat metabolism by regulating PPAR-*γ*. Present results thus strongly suggest that SM can be a potent therapeutic agent against TIIDM. 

## Figures and Tables

**Table 1:** List of primer sequences of RT-PCR with its amplicon size.

Gene	Accession number	Sequence forward primer 5′→3′	Sequence reverse primer 5′→3′	Product size
Glucokinase	NM_012565.1	AGTATGACCGGATGGTGGAT	CCGTGGAACAGAAGGTTCTC	139
Glut 2	NM_012879.1	CATTGCTGGAAGAAGCGTATCAG	GAGACCTTCTGCTCAGTCGACG	408
PEPCK	NM_198780	GTCACCATCACTTCCTGGAAGA	GGTGCAGAATCGCGAGTTG	84
Adiponectin	NM_144744.2	AATCCTGCCCAGTCATGAAG	CATCTCCTGGGTCACCCTTA	215
LPL	NM_012598.1	GAGATTTCTCTGTATGGCACA	CTGCAGATGAGAAACTTTCTC	276
Leptin	NM_013076.2	ACACCAAAACCCTCATCAAGA	GAAGGCAAGCTGGTGAGGA	184
SREBP-1c	XM_213329.4	GGCCTGCTTGGCTCTTCTC	GCCAGCCACAGCTGTTGAG	150
PPAR*γ*	NM_013124	GGATTCATGACCAGGGAGTTCCTC	GCGGTCTCCACTGAGAATAATGAC	156
Glut 4	NM_012751.1	GCCTTCTTTGAGATTGGTCC	CTGCTGTTTCCTTCATCCTG	457
*β*-ACTIN	NM_031144	CCTGCTTGCTGATCCACA	CTGACCGAGCGTGGCTAG	505

**Table 2:** Lipid profile of control and treated diabetic rats.

Groups	Control	DM	DM + EL	DM + SM	DM + metformin
Triglyceride^#^	55.86 ± 4.69	110.94 ± 14.32*	49.63 ± 6.37^a^	63.34 ± 7.86^b^	94.54 ± 19.31
Total cholesterol^#^	88.55 ± 7.28	123.26 ± 15.80*	95.15 ± 10.88^a^	86.75 ± 13.64^b^	106.78 ± 12.54
HDL-C^#^	55.86 ± 4.66	27.44 ± 8.27*	44.63 ± 5.49^a^	40.94 ± 6.77^b^	41.89 ± 4.69
LDL-C^#^	21.52 ± 1.93	73.63 ± 3.2*	40.59 ± 4.37^a^	33.14 ± 5.86^b^	45.99 ± 5.64^c^
VLDL-C^#^	11.17 ± 0.69	22.19 ± 4.76*	9.93 ± 1.3^a^	12.67 ± 1.01^b^	18.90 ± 2.31

^#^Units: mg/dL. Values are given as mean ± SEM from 6 rats in each group, *P < 0.005 compared to control and a, b, c compared to diabetic group.

## Abbreviations

DPP-4: Dipeptidyl peptidase-IV
EL: *Enicostemma littorale*
G-6-Pase: Glucose 6 phosphatase
GK: Glucokinase
GLP-1: Glucagon-like peptide-1
Glut 2: Glucose transporter 2
Glut 4: Glucose transporter 4
HGP: Hepatic glucose production
HMG CoA Reductase: 3-hydroxy-3-methyl-glutaryl-CoA reductase
HPTLC: High performance thin layer chromatography
IR: Insulin receptor
LPL: Lipoprotein lipase 1
MFO: Metformin
NA: Nicotinamide
NIDDM: Noninsulin dependent diabetes mellitus
OGTT: Oral glucose tolerance test
PEPCK: Phosphoenolpyruvate carboxykinase
PI(3)K: Phosphatidylinositide 3-kinases
PP_2_BS: Postprandial (2 hours) blood sugar
PPAR-*γ*: Peroxisome proliferator-activated receptor gamma
SM: Swertiamarin
SREBP-1c: Sterol regulatory element-binding protein-1c
STZ: Streptozotocin
TG: Triglycerides
TIIDM: Type II diabetes mellitus
WHO: World Health Organization.
